# Established, New and Emerging Concepts in Brain Vascular Development

**DOI:** 10.3389/fphys.2021.636736

**Published:** 2021-02-12

**Authors:** Ankan Gupta, Kevin R. Rarick, Ramani Ramchandran

**Affiliations:** ^1^Department of Pediatrics, Division of Neonatology, Developmental Vascular Biology Program, Children’s Research Institute (CRI), Medical College of Wisconsin, Milwaukee, WI, United States; ^2^Department of Pediatrics, Division of Critical Care, Children’s Research Institute (CRI), Medical College of Wisconsin, Milwaukee, WI, United States

**Keywords:** endothelial cell, brain, blood–brain barrier, neurovascular disease, neural stem cell, neuron, wnt

## Abstract

In this review, we discuss the state of our knowledge as it relates to embryonic brain vascular patterning in model systems zebrafish and mouse. We focus on the origins of endothelial cell and the distinguishing features of brain endothelial cells compared to non-brain endothelial cells, which is revealed by single cell RNA-sequencing methodologies. We also discuss the cross talk between brain endothelial cells and neural stem cells, and their effect on each other. In terms of mechanisms, we focus exclusively on Wnt signaling and the recent developments associated with this signaling network in brain vascular patterning, and the benefits and challenges associated with strategies for targeting the brain vasculature. We end the review with a discussion on the emerging areas of meningeal lymphatics, endothelial cilia biology and novel cerebrovascular structures identified in vertebrates.

## Introduction

Normal physiological function of the brain depends upon adequate supply of oxygen and nutrients. Cells in the brain rely on the brain vasculature for their supply of oxygen and nutrients, which are carried out by the macrovasculature (arteries and veins) and microvasculature (arterioles, capillaries, and venules). To meet the growing needs of the neural tissue, the brain concomitantly expands and remodels its vasculature. Alteration in the central nervous system (CNS) vascularization results in the progressive destruction of tissue, especially at the subventricular zone, which eventually leads to embryonic lethality (Raab et al., 2004). The brain vasculature develops exclusively via angiogenesis; a process of new vessel formation from existing established vasculature in contrast to *de novo* vasculogenesis, a process that involves assembly of vessels from endothelial precursor cells. In most tissues, vasculogenesis and angiogenesis processes work together to determine vessel expansion and remodeling (Risau, 1997; Louissaint et al., 2002). However in the brain, angiogenesis is the main process responsible for vascular development that results in 600 kilometer (∼372 miles) network of capillaries (Zlokovic, 2005). Brain endothelial cells (ECs) that line the vasculature are distinct from peripheral ECs in that they are in contact with more cell types (astrocytes and neurons) in addition to smooth muscle-like mural cells referred to as pericytes. Brain ECs also do not align to shear stress like the large caliber human umbilical vein ECs do and the shear stress encountered by the brain vessels are much different than those encountered by the peripheral vasculature (DeStefano et al., 2017). Further, the brain microvasculature is made up of capillaries and postcapillary venules which carries out its microcirculatory function. Brain capillaries are also structurally distinct from capillaries of the skin, lung, and liver in that they are continuous and non-fenestrated (lack of pores) with tight junctions that makes solute transport highly restrictive and regulated (Daneman and Prat, 2015; Zhao et al., 2015). Thus, the brain vasculature possesses distinct properties, which is suited to the unique cellular environment that the brain ECs reside in. In this review, we focus on the brain ECs and its interaction with cell types in the brain in both embryonic and adult life, the underlying mechanisms associated with this process, and the areas of emerging research in the brain.

## Brain Vascular Development: Lessons From Vertebrate and Mammalian Development

**FIGURE 1:**
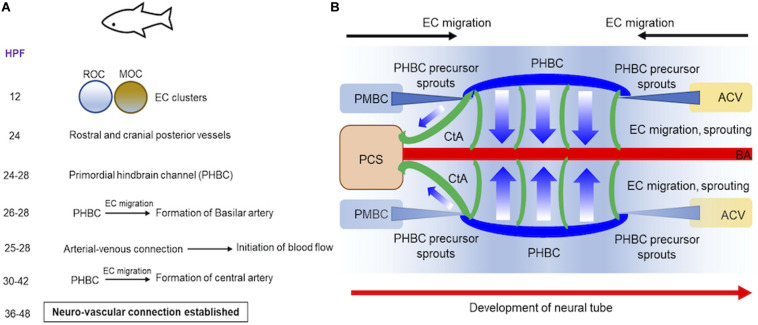
Progression of vascular development in brain of embryonic zebrafish. **(A)** A summary of the timeline of events demonstrating how two endothelial cell (ECs) clusters in rostral or midbrain organizing centers give rise to blood vessels in embryonic zebrafish is provided. **(B)** Schematics demonstrate how blood vessels in brain are formed at 24–48 hpf during embryonic development. Precursor cells sprout from PMBC or ACV that ultimately form PHBC (blue). ECs migration and sprouting occurs from PHBC to first establish BA (red) and subsequently CA (green). Direction of neural tube development is also shown (red arrow). **ACV,** Anterior cardinal vein; **BA**, basilar artery; **CA**, central artery; **ECs,** endothelial cells; **HPF,** hours post fertilization; **MOC,** midbrain organizing center; **PMBC,** primordial midbrain channel; **PHBC,** primordial hindbrain channel; **PCS,** posterior communicating segment; **ROC,** rostral organizing center.

**FIGURE 2:**
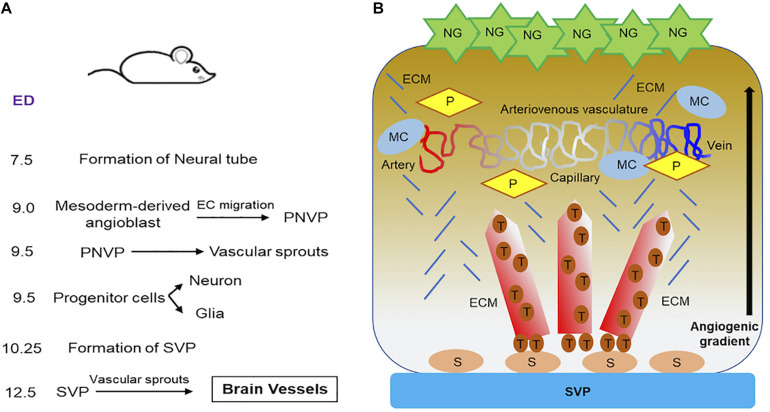
Progression of vascular development in brain of embryonic mouse. **(A)** A summary of the timeline of events showing how brain vessel is formed and synchronized to neural development in embryonic mouse is provided. **(B)** Schematics demonstrate how blood vessels in brain are formed and stabilized. During neurovascular development in embryonic mouse brain, neuroglia (NG) release proangiogenic factors. Vascular sprouts emerges from SVP as stalk cells differentiate into tip cells along the angiogenic gradient as established by neuroglia. These sprouting form the brain vessels, which are stabilized by the recruitment of ECM, mural cells and pericytes. The stable brain vessels eventually give rise to mature arteriovenous vasculature in the ventricular area of embryonic mouse hindbrain at E12.5 and beyond. **ECM**, extra cellular matrix; **ED,** embryonic days; **MC**, mural cell; **NG**, neuroglia; **P**, pericyte; **PNVP,** perineural vascular plexus; **S**, stalk cell; **SVP,** sub-ventricular vascular plexus; **T**, tip cell.

We will discuss our current understanding of brain vessel formation from studies in vertebrate zebrafish (Figures 1A,B) and mammalian mouse (Figures 2A,B) model systems (Tata et al., 2015). We focus on vascular development in the hindbrain and forebrain regions of the brain. Zebrafish, a freshwater fish from the Ganges river has contributed immensely to our understanding of vascular development primarily because: embryos develop fairly rapidly *ex vivo* and are transparent in embryonic stage, and genetic manipulation is relatively straightforward with injection of RNA, DNA and oligonucleotides feasible at the 1-cell stage. The genetic engineering methods facilitated the development of tissue-specific fluorescent reporter gene expressing transgenic lines, which when combined with confocal and 2-photon microscopy, provided deep insights into the vascular assembly processes in the developing brain. Early studies on mutants identified in the ethylnitrosurea (enu)-induced mutagenesis screens reported *violet beauregarde* (*vbg*) (causative allele: *activin-receptor-like kinase, ALK1*), which showed increased ECs numbers in the brain at 2–2.25 days post fertilization (dpf) (Roman et al., 2002). Using the vascular-specific transgenic reporter line (*etv2*:GFP), time-lapse imaging revealed onset of two major clusters of cells in the 12 hour post fertilization (hpf) embryonic brain namely the rostral organizing center and the midbrain organizing center. By 24 h, these two cell clusters give rise to the most rostral and posterior cranial vessels, respectively (Proulx et al., 2010) (Figures 1A,B). Subsequently, using two different vascular transgenic lines (*kdrl*:GFP & *fli1a*:EGFP), additional details of the hindbrain vascular patterning process were identified (Fujita et al., 2011; Ulrich et al., 2011). Two sets of precursor cells (24–28 hpf), one from the anterior end, primordial midbrain-derived and second from posterior end namely anterior cardinal vein-derived, migrate and form the primordial hindbrain channels (PHBCs) (Ulrich et al., 2011) (Figure 1B). At 26–28 hpf, basilar artery, the major blood vessel that supplies the hindbrain is formed by medial sprouting and migration of ECs from the bilateral pair of PHBCs veins (Fujita et al., 2011) (Figures 1A,B). A second wave of sprouting (30–42 hpf) occurs from PHBCs that gives rise to central arteries (CtAs) (Figure 1B), which penetrate and vascularize the hindbrain at the rhombomere (segment of the developing neural tube) boundaries (Fujita et al., 2011; Ulrich et al., 2011). Flow, which commences between 25 and 28 hpf in the developing zebrafish brain has been implicated as a critical feature that ensures the proper formation of arterial-venous connection and the establishment of a functional circulatory loop (Bussmann et al., 2011). Between 36 and 48 hpf, a subset of hindbrain vessels has aligned proximally to neuron clusters and axon tracts suggesting cross-communication between these cell types during development (Ulrich et al., 2011). Moving posteriorly, the integration of the vascular systems between the hindbrain and spinal cord was determined using time lapse imaging in 3–4 dpf *fli1a*:EGFP & *fli1a*:nEGFP lines (Kimura et al., 2015).

In the zebrafish forebrain, much of our knowledge of vascular patterning has emerged from high-resolution time-lapse imaging of the palatocerebral artery (PLA), which forms via angiogenesis (Lenard et al., 2013). PLA runs along the base of the forebrain and connects two cranial internal carotid artery that encapsulate the optic cup on each side of the embryonic head. PLA forms through fusion of two lumenized angiogenic sprouts, and blood flow influences the transcellular lumen formation. *VE-cadherin* (a pivotal EC junction marker) plays a critical role in the initial steps of the vessel fusion process (Lenard et al., 2013). Vessel anastomosis (fusion) and establishment of ECs polarity are coordinated processes, which occur in a stepwise manner.

The brain development in mouse (Figure 2A) begins as early as embryonic day 7.5 (E7.5), when the formation of neural tube begins. At E9.5, dorsoventral patterning of neural tube progenitors is established (Dessaud et al., 2008). During this time, neurons and glial cells differentiate from progenitor cells and begin migrating, an event that coincides with the development of the brain vasculature. Vascularization of spinal cord and brain is initiated before birth through the angiogenic sprouting networks, specifically, the perineural vascular plexus (PNVP) and the periventricular plexus (PVP) (Ruhrberg and Bautch, 2013). PNVP arises from mesoderm-derived angioblasts (endothelial precursor cells) and covers the entire CNS by E9.0 (Hogan et al., 2004; Engelhardt and Liebner, 2014) (Figure 2A). Half a day later, the mouse hindbrain vascularization begins. Vascular sprouts emerge from PNVP and grow in a radial direction toward the ventricular zone in the direction of the neural progenitor cell location, which are thought to secrete vascular growth factors that stimulate the radial migration of ECs (Fantin et al., 2010). At E10.25, radial vessels extend at 90° angle and parallel to the hindbrain surface when they intersect with neighboring radial vessels and anastomose to become the sub-ventricular vascular plexus (SVP). This anastomosis process is facilitated by macrophages (precursors of microglia) (Fantin et al., 2010). At E12.5, an extensive vascular network has emerged from SVP, which sprouts and penetrates deep into the brain based on cues from neural glial cells (Figure 2B). For additional details on the brain anatomy and vessel location in mouse brain, we refer the reader to a detailed review on this subject (Puelles et al., 2019).

The mouse forebrain vascularization commences at E9.5, which also occurs from the PNVP and progresses across the entire rostro-caudal axis in a ventrolateral or dorsomedial direction. At E10.0, the ventral forebrain is vascularized by PNVP while the dorsal region is largely avascular. Intriguingly, vasculature in the dorsal forebrain does not arise from dorsal PNVP but instead, it is derived from the SVP of the ventral compartment (Vasudevan et al., 2008). By E11.0, SVP is formed in both ventral and dorsal areas of the forebrain including the dorsal medial wall region. In addition to PNVP, vascularization of the embryonic forebrain also occurs from the PVP. The PVP vascularization process is regulated by EC-derived transcription factors (Vasudevan et al., 2008). EC migrates from the surrounding PNVP into the neuroepithelium and initiates CNS vascularization, and also migrate from the pial surface toward the subventricular zone. As ECs migrate into these avascular regions, they adopt specific phenotypes (Haigh et al., 2003; Mancuso et al., 2008). As the neural tissue expands, the blood vessels grow into vast networks and remodels into arterial and venous vasculature.

The nascent brain vasculature continues to develop with cues from surrounding brain resident cells, and is stabilized via the recruitment of mural cells, and establishment of extracellular matrix (Jain, 2003). This growth and maturation of the brain vasculature coincides with the generation of different neural cell types and establishment of the neural circuit (Vasudevan et al., 2008; Ulrich et al., 2011; Tata et al., 2016). The timing of this vascular maturation is critical toward serving the metabolic need of developing neural tissue and the expansion of various neural cells in the brain (Knobloch and Jessberger, 2017). Thus, coordination of the neural and vascular development processes in the brain is necessary and suggests that crosstalk between these two cellular systems are critical for physiological brain development.

## Evolution and Genetic Signature of a Brain Endothelial Cell – Insights From Single Cell Sequencing Data Analysis

The origin of brain ECs in mammals is not well understood and understudied. The long-standing hypothesis in the field is that brain-derived ECs are unique with respect to distinct transcriptome and gene expression signatures, and function. Clues for brain ECs origin have emerged from studying the expression of a transcription factor Sox2, which is a key regulator of neuronal differentiation and brain development (Amador-Arjona et al., 2015). Flk1^+^ Sox2^+^ ECs were identified adjacent to the developing brain cells that only express Sox2 at E10.5 and E14.0 (Bostrom et al., 2018). At E12.5, Sox2^+^ VE-cadherin^+^ EC sub-population were observed, which was absent at E18.5 upon development of the vasculature. These data collectively suggest that Sox2*-*marked cells are undergoing sub-selection for vascular lineage specification, leading to progressive temporal expression of vascular markers Flk1 and VE-cadherin. Knowledge from these initial observations has expanded further with the advent of single cell RNA sequencing technologies (scRNA-seq) (Picelli, 2017; Potter, 2018). Profiling of individual brain ECs suggested great heterogeneity in this population and extensive molecular changes during embryonic development (Hupe et al., 2017). Using scRNA-seq method, 15 distinct cell sub-types of mouse brain ECs have been observed compared to 17 distinct cell sub-types of mouse lung ECs (He et al., 2018). Not surprisingly, mouse embryonic brain ECs show more features related to BBB differentiation, while post-natal brain ECs reveal distinct relationships between cell types (for example, arteries and tip cells, veins, and mitotic cells) (Sabbagh et al., 2018). When EC-specific translating ribosome affinity purification (EC-TAP) was combined with scRNA-seq methodology, additional low abundance transcripts were revealed (Cleuren et al., 2019), and marked differences across vascular beds was observed when host was challenged with stress factors (lipopolysaccharides). The scRNA-seq studies also revealed that organ-specific ECs typically show expression patterns that mimic the site that they reside in Jambusaria et al. (2020). For example, brain ECs express synaptic vesicle genes or cardiac ECs express contractile genes. This tissue-specific heterogeneity of EC expression pattern is also conserved during disease conditions such as inflammation. Further, brain- and liver-specific ECs cluster strongly by tissue of residence while others, adipose- and heart-specific ECs overlap with ECs from other tissues (Paik et al., 2020).

In a comprehensive study of >32,000 single EC transcriptomes from 11 mouse tissues revealed some interesting insights (Kalucka et al., 2020). First, ECs from somewhat unsuspected pairs of tissue (brain/testis, liver/spleen, small intestine/colon, and skeletal muscle/heart) show partially overlapping gene expression. Second, tissue rather than vessel type contributed to the EC heterogeneity. Third, capillary ECs in a tissue are more heterogenous in gene expression than arterial, venous, and lymphatic ECs in that tissue. Fourth, transcriptomes of metabolic gene products showed distinct patterns across vessel types in a given tissue and was reflective of the respective tissue function. Additional scRNA-seq studies also revealed during aging that hippocampal brain capillary ECs undergo the greatest transcriptional changes, upregulate innate immunity and oxidative stress response pathways compared to hippocampal arterial or venous brain ECs (Chen et al., 2020b). Further, senescent EC numbers increases by 10% in the mouse cerebral microcirculation (Kiss et al., 2020). Thus, transcriptional age of brain ECs are sensitive to age-related circulatory cues (Chen et al., 2020b). Another noteworthy connection that emerged from scRNA-seq analysis is the one between tip cells and aortic ECs (Sabbagh et al., 2018). Tip cells are front line cells of the plexus and act as “sensor” of growth factor gradients, and rarely proliferate. Stalk cells, which are located behind the tip cells, proliferate, form the vascular lumen and help extend the length of the growing sprout (Gerhardt et al., 2003; Ridley et al., 2003; Mancuso et al., 2008). Brain capillary EC clusters identified by scRNA-seq are enriched for cells expressing catecholamine DOPA Decarboxylase (Ddc), which was previously reported to express in aortic ECs (Sorriento et al., 2012). Similarly, CXCR4 receptor (tip cell marker) (Strasser et al., 2010) was observed in brain-derived EC clusters, and its ligand CXCL12 was enriched in the arterial EC cluster. These data collectively suggest that a communication signal may exist between endothelial sprouting tip cells and arterial ECs in the brain. These examples highlight the ability of scRNA-seq method to provide unexpected insights and spur new areas of EC biology.

## Endothelial Cell and Its Effect on Neural Stem Cells in the Developing and Adult Brain

In the embryonic CNS, the vascular and neural compartments develop concomitantly (Karakatsani et al., 2019). The mammalian neocortex is defined by six layers of neurons that develops from a single layer of neuroepithelial cells called radial glial cells (RGs), also referred to as neural stem cells (NSCs). RGs undergo extensive symmetric division to expand, followed by differentiation into neurons or basal progenitors, and finally symmetric division to generate post-mitotic pyramidal neurons, which migrate to attain their terminal position in the cortex. All these processes occur between E10.5 and E17.5 in the mouse embryonic brain (Gotz and Huttner, 2005). The early embryonic brain is hypoxic because of lack of vasculature, and in this hypoxic microenvironment, NSCs proliferation is abundant (Mohyeldin et al., 2010). Positional proximity of developing vasculature in brain sets the microenvironment conducive for the expansion of neural progenitors (Javaherian and Kriegstein, 2009; Nie et al., 2010). Interestingly, the relief of hypoxia by angiogenesis promotes NSCs differentiation (Lange et al., 2016b). Premature neuronal differentiation at the expense of reduced self-renewal of NSCs occurs in a reduced angiogenic state (Tata et al., 2016). Thus, the vasculature that develops from the PVVP (E8.5–E10.0) and PNVP (E11.0–E12.5) provide nourishment to growing stem cell niches and balance the expansion vs differentiation of NSCs. ECs effect on NSCs and the reverse effect of NSCs on ECs have been well documented. For example, ECs when co-cultured with embryonic NSCs, promote stem cell maintenance through unknown paracrine factors (Gama Sosa et al., 2007; Vissapragada et al., 2014), and enhance NSC survival and preserve their pluripotency (Lowry et al., 2008). On the contrary, conditioned media from RGs decreases brain ECs proliferation (da Silva et al., 2019), promotes migration and formation of vessel-like structures *in vitro* (Siqueira et al., 2018). Also, in an autopsy of telencephalon from 22-week old human embryo, a defined Gfap^+^ Cx43^+^ CXCL12^+^ RG population appeared to establish physical contact and interaction with angiogenic-activated (CD105^+^) ECs (Errede et al., 2014). These specialized contacts, recognizable on both perforating radial vessels and growing collaterals, appeared as CXCL12-reactive. In absence of RG cells, a significant reduction has been observed of cortical thickness and the regression of nascent brain vessels, *via* the inhibition of EC-specific Wnt signaling in a contact and stage-dependent manner (Ma et al., 2013). In the adult, similar to embryonic stage, the vasculature is required not only for transporting oxygen and nutrients but also for trophic support of the neuronal compartment (Licht and Keshet, 2015; Ramasamy et al., 2015). The ECs are located adjacent to self-renewing multipotent NSCs populations in sub-ventricular zone (SVZ) and sub-granular zone (SGZ) both in the adult (Gage, 2000; Alvarez-Buylla and Lim, 2004). ECs indeed promote NSCs proliferation and differentiation (Han et al., 2015) via secretion of VEGF-C that act on its cognate receptor VEGFR-3 expressed on NSCs. Further, the EC’s role in maintaining NSCs quiescence was suggested as cell-cell contact mediated, with ephrinB2 and Jagged1 identified as molecules that were responsible for this process (Ottone et al., 2014). Thus, ECs and NSCs depend on the other for sustenance during embryonic and adult stages.

## Signaling Molecules: Focus on Emerging Wnt-β Catenin Signaling Pathway to Shape Brain Vascular Development

**FIGURE 3:**
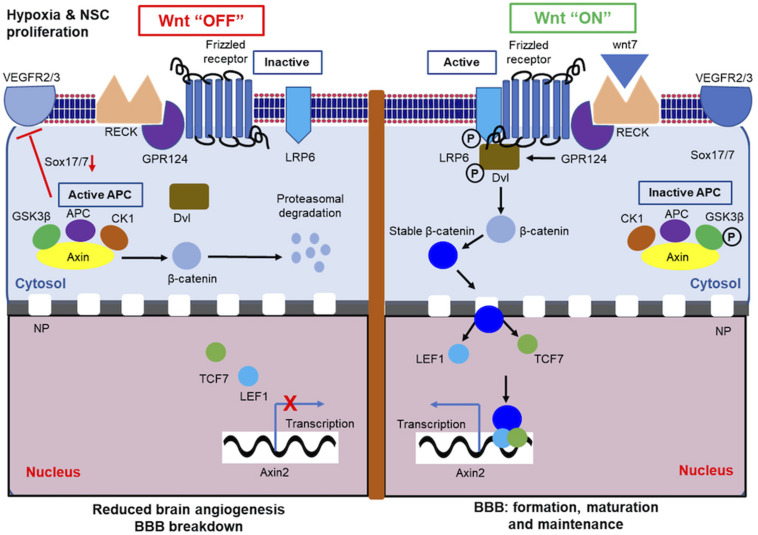
Canonical Wnt pathway in brain endothelial cells (ECs). In absence of its cognate ligand, canonical Wnt pathway remains inactive or “OFF” state. In the OFF state, cytosolic axin protein complex (APC) prevents stabilization of β-catenin and promotes its proteasomal degradation. In the Wnt “OFF” condition, axin indirectly downregulates VEGFR2 or VEGFR3 by inhibiting Sox17/7 and abrogates CNS angiogenesis. In active or Wnt “ON” state, Wnt binds to its receptor located in the surface of brain ECs, which causes the stabilization of cytosolic β-catenin. Stable β-catenin is then translocated to nucleus to promote transcription of downstream target genes. **P,** phosphorylation; **NP,** nuclear pore. In brain ECs Wnt OFF state, hypoxic condition is ensued, which in turn influences neural stem cell (NSC) proliferation.

A survey of the literature for signaling pathways impacting the brain vasculature formation shows that VEGF, TGF-β, and Wnt signaling are repeated themes that emerge. Both VEGF and TGF-β signaling pathway in the context of brain development have been extensively reviewed elsewhere (Rodriguez-Martinez and Velasco, 2012; Lange et al., 2016a). In this review, we emphasize Wnt signaling and its role in brain vascular patterning. Wnt signaling (Figure 3) is one of the pivotal evolutionarily conserved networks that orchestrates cell–cell communication during the embryonic development of multicellular organisms (Clevers, 2006; MacDonald et al., 2009; Clevers and Nusse, 2012). The Wnt signaling pathway directs cell proliferation, cell polarity, and cell fate determination during embryonic development (Logan and Nusse, 2004). Mutations in the Wnt pathway are often linked to congenital defects (Clevers, 2006). The developmental importance of Wnt protein was first demonstrated in larval development of *Drosophila*, where Wnt1 homolog was shown to regulate segment polarity (Nusslein-Volhard and Wieschaus, 1980). The study on developmental significance of Wnt signaling cascade was further extended in *Drosophila* as well as in *Xenopus* (McMahon and Moon, 1989; Siegfried et al., 1992; Noordermeer et al., 1994; Peifer et al., 1994). Once gastrulation is commenced, Wnt/β-catenin signaling activates a defined transcriptional program that directs anteroposterior axis development, leading to the development of head structures and formation of tail (Green et al., 2015). In the last four decades, “canonical” Wnt signaling has been extensively studied and emerged as a major Wnt pathway that regulates key developmental gene expression programs (MacDonald et al., 2009). In the absence of ligand Wnt (“Wnt switch off”), the cytoplasmic β-catenin protein is degraded by an Axin protein complex, which includes adematous polyposis coli gene (*APC*), casein kinase 1 (*CK1*), and glycogen synthase 3 (*GSK3*). CK1 and GSK3 phosphorylates specific amino acid residues in β-catenin in a specific sequence, which leads to its recognition by β-Trcp, an *E3* ubiquitin ligase subunit that targets β-catenin protein for proteasomal degradation (Figure 3). In the presence of ligand Wnt, it binds to a seven pass-transmembrane Frizzled (Fz) receptor and co-receptor low-density lipoprotein receptor-related protein 6 (LRP6) complex leading to recruitment of the scaffolding protein Dishevelled (Dvl) resulting in LRP6 phosphorylation. This results in inhibition of Axin-mediated β-catenin phosphorylation leading to β-catenin stabilization, accumulation of β-catenin in the cytoplasm, followed by entry into the nucleus. In the nucleus, β-catenin engages with DNA-bound TCF transcription factors (Behrens et al., 1996; Molenaar et al., 1996) to activate Wnt target genes (“Wnt switch on”) (Lee et al., 2009; Hikasa et al., 2010) (Figure 3). In the “Wnt switch off” condition, TCFs interact with specific transcriptional repressors (Cavallo et al., 1998; Roose et al., 1998) preventing the gene transcription. *Axin2* gene is a global transcriptional target of Wnt and is therefore considered a “generic” index of Wnt pathway activity (Lustig et al., 2002).

In mammals, Wnt signaling is facilitated by 19 ligands (MacDonald et al., 2009), targets several genes (Vlad et al., 2008), and the effects are cell or context-specific (Logan and Nusse, 2004). Apart from canonical Wnt signaling cascade, there are two additional pathways that are also known to be activated following Wnt receptor activation (Clevers, 2006), a noncanonical planar cell polarity (PCP) pathway (Seifert and Mlodzik, 2007; Wang and Nathans, 2007), and a Wnt/Ca^2+^ pathway (Kohn and Moon, 2005). In the non-canonical pathway, Wnts bind to Fz receptors and activate Dvl, independent of GSK-3β or β-catenin. Other signaling proteins involved in the non-canonical pathway activation include small GTPases, the heterotrimeric G proteins, and, in some cases, *C*-Jun *N*-terminal kinase (Mlodzik, 2002; Fanto and McNeill, 2004; Montcouquiol et al., 2006). In the second non-canonical pathway, certain combination of Wnts and Fzs can activate intracellular calcium, which in turn induces calcium-calmodulin-dependent kinase (CAMKII) and protein kinase C (Moon et al., 1993; Du et al., 1995; Sheldahl et al., 1999; Kuhl et al., 2000). Thus, Wnt signaling has several ways to trigger signaling cascades associated with specific phenotypic readouts, which makes it a prime candidate for fine tuning of brain angiogenesis in the developing and adult vasculature.

Several Wnt family members are expressed in the neural tube, which coincides with neural tube angiogenesis (Parr et al., 1993). Enrichment of high mobility group transcription factors Lef1 and Tcf7 are indicators of active canonical Wnt signaling in brain ECs (Cadigan and Waterman, 2012; Sabbagh et al., 2018). Impaired endothelial β-catenin signaling in experimental animal model results in abrogated ECs proliferation and sprouting that ultimately causes hypo-vascularization of the brain (Martowicz et al., 2019). ECs with and without β-catenin formed tip cell suggesting that endothelial β-catenin is not absolutely needed for tip cell formation. However, the tip cell’s ability to compete for the tip cell position was compromised. Notably, impaired endothelial β-catenin signaling abrogated the expression of the VEGFR2 (tip cell selection marker) and VEGFR3 in brain microvessels but not in the lung endothelium, suggesting that the β-catenin-EC-specific functional implication are specific to brain ECs (CNS) compared to peripheral ECs (non-CNS) (Martowicz et al., 2019). Several lines of evidence support the theory of restriction of Wnt signaling to the CNS versus non-CNS tissue. Spatial distribution of Wnt ligands in CNS and non-CNS tissues were investigated using EC-specific (*Tie2-Cre*) mouse line that was crossed to Wnt reporter (*R26-Tcf/Lef-LoxPSTOPLoxP-H2B-GFP-6xMYC*) mice (Sabbagh et al., 2018). In this model, Cre recombinase enzyme-mediated excision of a *LoxP*-flanked transcription stop cassette allows for visualization of active Wnt signaling in ECs wherein the multimerized TCF/LEF motifs, together with a minimal promoter drives the expression of a nuclear-localized histone *H2B-GFP-6xMYC* fusion protein (Cho et al., 2017a). The nuclear accumulation of LEF1, which is both a mediator and a marker of canonical Wnt signaling was confined to the CNS and perineural ECs. This observation implies that the canonical wnt signaling is active in ECs of CNS but not elsewhere. In a second-independent approach (Daneman et al., 2009), a Wnt reporter *TOP-GAL* transgenic mice was used that expresses the *lacZ* transgene under the control of *Tcf* promoters, a downstream effector of Wnt signaling. Thus, *LacZ* expression occurs in cells where canonical Wnt/catenin signaling is activated. Activated Wnt signaling was co-localized with the transgenic EC marker (*Tie2-*GFP) only in the CNS, but not in peripheral tissues. To implicate β-catenin function in Wnt signaling in ECs, an EC-specific β-catenin knock-out mice (*Tie2 cre*^+/^*^–^ β-catenin^flox/flox^*) was generated. Normal vascular pattern in non-neural tissues was retained while major vascular defects were observed in the CNS of all knockout mice. No capillaries were formed throughout the developing forebrain and the PNVP was significantly thickened. In terms of Wnt signaling and its role in BBB, EC-specific β-catenin activation *in vivo* was necessary for formation and maintenance of BBB, and enhanced barrier maturation, while inactivation of this pathway contributed to BBB breakdown (Liebner et al., 2008).

To date, how brain ECs respond and react in the brain microenvironment is not fully understood but recent studies are beginning to shed some light on this process. In the first zebrafish study, GPI-anchored MMP inhibitor Reversion Inducing Cysteine Rich Protein with Kazal Motifs (RECK) was identified as critical for cerebrovascular development and promotes canonical Wnt signaling (Ulrich et al., 2016). A second zebrafish study suggested that an orphan G-protein coupled receptor (GPCR) Gpr124 along with RECK worked together as integral components of the Wnt-specific signaling complex to facilitate brain angiogenesis (tip cell development) and dorsal root sensory neurogenesis (Vanhollebeke et al., 2015). This GPR124-RECK-WNT signaling axes was also later confirmed in the mouse CNS angiogenesis (Cho et al., 2017b). GPR124, an orphan GPCR has been previously reported by several groups as essential for CNS vascularization (embryonic and adult) (Kuhnert et al., 2010; Anderson et al., 2011), establishment of the BBB (Cullen et al., 2011; Zhou and Nathans, 2014), and BBB integrity (Chang et al., 2017). Biochemical studies reveal that GPR124 through its ectodomain binds to RECK, and RECK binds to Wnt7A and 7B ligands but not Wnt3A ligand (Vallon et al., 2018). Further, RECK binds with low micromolar affinity to the disordered region of Wnt7 ligand (Eubelen et al., 2018). This interaction leads to Wnt receptor Frizzled signaling, which is dependent in part on the interaction between GPR124 and Dvl, a downstream phosphoprotein from the Frizzled receptor (Figure 3). Thus, RECK is a selective Wnt receptor that mediates GPR124/Frizzled/LRP-dependent canonical Wnt-β-catenin signaling. The extracellular interactions are partly associated with controlling the bioavailability of Wnt ligand for signaling (Eubelen et al., 2018; Vallon et al., 2018) and is a key regulatory step in this mechanism. Intracellularly, GPR124 contains a PDZ domain that is responsible partly for Wnt7-stimulated β-catenin signaling in brain ECs (Posokhova et al., 2015). Taken together, several studies have made inroads into our understanding of the genetic and biochemical mechanisms associated with Wnt signaling in the brain vasculature. Collectively, these studies imply that canonical Wnt-β-catenin signaling is active, functions in a cell autonomous manner in brain ECs, specific to the CNS, and facilitates BBB formation and maintenance through specific protein–protein interactions.

## Emerging Topics, Future Directions, and Perspectives

Thus far, we have extensively discussed the brain ECs and the underlying mechanism that is involved in their inception and development. In this section, we will discuss emerging topics of interest to the brain vascular field including meningeal lymphatic ECs, the role of brain microvascular EC cilia to vascular stability and the discovery of a new cerebrovascular structure in vertebrates.

## Endothelial Cells of the Meningeal Lymphatic System

The recent discovery of a meningeal lymphatic vascular system in the dura mater adds to the ongoing controversy surrounding brain waste clearance that includes glymphatic, paravascular, and perivascular pathways (Szentistvanyi et al., 1984; Iliff et al., 2012; Morris et al., 2016; Bacyinski et al., 2017). While there is evidence to support the existence of each of these distinct routes, the total contribution of each pathway to waste clearance under physiologic and pathologic conditions remains to be determined. In this section we will briefly discuss the development of meningeal lymphatics, provide a comparison with peripheral lymphatics, and present their role in emerging areas of interest. Additional, in-depth reviews have previously been published on origins and development of lymphatic ECs and meningeal lymphatic vessels (Balint et al., 2019; Gutierrez-Miranda and Yaniv, 2020). Studies in mice have determined that peripheral lymphatic vessels develop out of venous ECs from the common cardinal vein. Around embryonic day E9.5 to E10, venous ECs start to express prospero homeobox protein 1 transcription factor (PROX1) (Antila et al., 2017). After this, vascular endothelial growth factor receptor 3 (VEGFR3)^+^ lymphatic EC progenitors begin to sprout from the common cardinal vein to develop the first peripheral lymphatic plexus. Additional sprouting in response to the paracrine action of VEGF-C expands the lymphatic vascular tree (i.e., lymphangiogenesis). The identification of these specific lymphatic endothelial cell (LEC) markers allowed for the discovery of the meningeal lymphatic system. CNS lymphatic vessels positive for the classic lymphatic EC markers PROX1, VEGFR3, and podoplanin (PDPN) were recently identified using whole-mount preparations of dissected mouse brain meninges (Aspelund et al., 2015; Louveau et al., 2015). With this technique, Louveau et al. (2015) demonstrated the existence of independent vessel structures that run parallel to the dural sinus veins within the meningeal layer. These vessel structures were confirmed to be lymphatic vessels since they were lined with cells positive for lymphatic vessel endothelial hyaluronan receptor 1 (LYVE1) and were not connected to the cardiovascular circulation as they were not labeled by intravenous injection of fluorescent lectin. Furthermore, Aspelund et al. (2015) demonstrated the entirety of the network of lymphatic vessels within the CNS and surrounding meninges by analyzing *Prox1-*GFP and *Vegfr3*+*/LacZ* reporter mice. While no lymphatic vessels were observed on the brain parenchyma or the pia mater, lymphatic vessels were visualized along the superior sagittal sinus, the transverse sinus, the rostral rhinal veins, the middle meningeal artery, as well as exiting the skull along the meningeal portions of the pterygopalatine artery and cranial nerves (Aspelund et al., 2015). Like peripheral lymphatic vessels, the meningeal lymphatics also function to transport fluid and immune cells allowing for waste clearance and immune surveillance of the CNS. Unlike ECs of the blood brain barrier, meningeal LECs are characterized by fenestrated endothelium and absence of a basal membrane. Also, the meningeal lymphatics do not have valves to prevent back flow like their peripheral counterparts with the noted exception of some vessel segments located near the base of the skull (Aspelund et al., 2015). RNAseq analysis comparing meningeal LECs to those obtained from peripheral lymphatics of the diaphragm and skin showed high similarity in LEC-specific gene sets. However, gene set enrichment analysis suggested that the specific microenvironment of the LECs influences cell phenotype as multiple pathways related to extracellular matrix, focal adhesion, angiogenesis, and response to endogenous and exogenous stimuli were uniquely altered (Louveau et al., 2018). This theme of tissue environment influencing LECs gene expression is reminiscent to that observed for blood ECs in brain and other tissues described earlier in this review. Efficient brain waste clearance of molecules such as amyloid beta and tau is considered an important mechanism to alleviate neuronal injury and degeneration in Alzheimer’s disease and after traumatic brain injury (Iliff et al., 2012, 2014; Peng et al., 2016; de Leon et al., 2017). In addition to waste molecules, meningeal lymphatics have a role in regulating CNS immune responses as a route for antigen and immune cell drainage and for clearing red blood cells from the subarachnoid space after hemorrhagic stroke (Louveau et al., 2018; Chen et al., 2020a). Interfering with the normal clearance function of the meningeal lymphatic pathway has been shown to cause cognitive impairment in mice, increase pathology in experimental autoimmune encephalomyelitis (EAE) models of multiple sclerosis, and increase edema and infarction volume in an ischemic stroke rat model (Si et al., 2006; Radjavi et al., 2014; Louveau et al., 2018). Importantly, CSF drainage via meningeal lymphatic vessels to the deep cervical lymph nodes has been demonstrated in humans and non-human primates using contrast enhanced MRI scans further suggesting that meningeal lymphatic pathways are an emerging area of important clinical relevance for multiple CNS injury and neurodegenerative diseases (Absinta et al., 2017; Eide et al., 2018; Wu et al., 2020).

## Brain Microvascular Endothelial Cell Cilia

Growing evidence in the literature suggests that a microtubule-based cell organelle called cilia that projects from the apical surface of ECs into the lumen (Goetz et al., 2014), is thought to function as a cellular antenna and a central processing unit (Malicki and Johnson, 2017) and signaling center (Goetz and Anderson, 2010). Recently, endothelial cilia is thought to play an important role in brain vascular barrier function (Ma and Zhou, 2020), which is an emerging area of research in brain vascular biology. Cilia are found in most cells (Goetz and Anderson, 2010), and defects in cilia are often collectively referred to as “ciliopathies.” In ECs, cilia are widely considered as a flow sensor (Nauli et al., 2008; Egorova et al., 2012) and often reported as mechanosensors (Luu et al., 2018) wherein they convert mechanical input (flow-mediated) into chemical signaling inside the cell. The prevailing theory is that upon blood flow, cilia bends at an angle of 45° (Goetz et al., 2014), which triggers the release of calcium into the cells (Nauli et al., 2008; Ando and Yamamoto, 2013; Goetz et al., 2014), and subsequent cellular signaling effects (Hierck et al., 2008). Cilia is often considered a low-flow sensor (Goetz et al., 2014; Vion et al., 2018), and upon high flow, they have been shown to be lost from macrovessel (Iomini et al., 2004). Cilia expression and function in brain vessels have not been comprehensively investigated, until recently. We showed using confocal imaging of zebrafish vasculature that cilia are found in brain ECs prior to flow, during flow, and post establishment of high flow (Eisa-Beygi et al., 2018). We also found cilia in vasculogenic, and angiogenic vessels in the brain, and during various distinct processes associated with the vessel growth such as sprouting, anastomosis and lumen formation. Thus, these data collectively suggest that brain EC-cilia have functions beyond just sensing flow (Norris and Santoro, 2018). Knocking out or knocking down proteins in cilia causes the brain vessels to rupture followed by intracranial hemorrhage (Kallakuri et al., 2015; Eisa-Beygi et al., 2018; Pollock et al., 2020). These hemorrhages are exacerbated by enhanced shear stress (Eisa-Beygi et al., 2018). Similarly, polycystic kidney mutant fish and *ciliary intraflagellar transport (IFT)* protein mutant fish show intracranial vessel hemorrhage (Kallakuri et al., 2015; Pollock et al., 2020). Re-expressing of the IFT protein in the brain ECs rescued the hemorrhage phenotype thus arguing for a cell autonomous function for cilia in vascular stabilization (Eisa-Beygi et al., 2018). Similarly, mouse mutants *Ift172* (Gorivodsky et al., 2009) and *Ift122* (Cortellino et al., 2009), both show cranial neural tube defects and bleeding, and ECs isolated from *Ift88* mice with polycystic kidney disease show higher permeability to dextran (Jones et al., 2012). In addition to its role in promoting vascular stability through ECs junctional integrity, EC-cilia has also been recently implicated to recruit support cells such as mural cells that stabilizes the brain vasculature (Chen et al., 2017). Thus, we hypothesize that cilia role in vascular barrier formation and the underlying mechanisms associated with cilia-mediated vascular stability are perhaps the next areas of brain vascular integrity research. Given the propensity for cerebrovascular incidents in several patient populations including sickle cell disease (Hirtz and Kirkham, 2019), preeclampsia (Miller, 2019) and others, it will not be surprising if brain EC-cilia emerges as a key signaling center that contributes to the cerebral vessel pathogenesis. Finally, in relation to lymphatics ECs, it is unknown whether cilia are expressed in LECs and if so, what their potential function is? These and other emerging questions will keep scientists from numerous multi-disciplines busy.

## New Transient Cerebrovascular Structure in Vertebrates

In a recent zebrafish study (Kugler et al., 2019), using time series light sheet microscopy of brain vessels in 3 days old fish, the authors observed a spherical EC membrane structure that is transient in nature and protrudes from the cerebral vessel. This structure dubbed “*kugeln*” (German for sphere) was observed as early as 3 days post fertilization (dpf), and as late as 28 dpf. On an average, *kugeln* was observed to exist for 23 min, formed in the absence of flow, and does not communicate with vessel lumen. *Kugeln* also contains little to no cytoplasm, no nuclei but is filled with nitric oxide. Further, *kugeln* does not interact with brain lymphatic ECs, or with macrophage cells in the brain nor was it observed in peripheral trunk vessels. Inhibition of VEGF signaling or Wnt signaling dysregulation increases *kugeln* formation, and inhibition of actin polymerization, myosin II or Notch signaling decreases *kugeln* formation. The obvious question of *kugeln*’s role and function remains unknown, and a congruent *kugeln* structure in mammalian brain vessels has not been identified to date. The presence of such dynamic structures in cerebral vessels emphasizes the point that so much is still unknown regarding how brain vessels pattern. These and other such discoveries will bring fresh and novel perspectives to the field of brain vascular biology.

## Conclusion

Considerable progress in our understanding of the developing brain vascular patterning process has been made in vertebrates, and more is yet to come especially at the mechanistic level. In this review, we discussed the contributions of the zebrafish and the mouse model systems to the brain vessel patterning process. The contribution of various cell types in the brain to the ECs-driven vascular patterning process is an untapped area of research. Mechanisms associated with vasculogenesis, angiogenesis and the origin of brain ECs are beginning to emerge. Single cell sequencing technology is providing a framework for new questions such as the similarity of ECs between brain and testis. Details related to various molecules that participate together to mechanistically control Wnt signaling in brain vascular development is coming to focus. Finally, meningeal lymphatics, brain EC-cilia and a new brain-specific vascular structure *kugeln* are emerging areas of research that will offer new insights into brain vascular patterning and homeostasis. These basic science studies are likely to contribute to better understanding of vascular compromise states in several clinical conditions that afflict the brain.

## Author Contributions

AG partly conceptualized, wrote, and edited the manuscript. KR assisted in writing parts of the manuscript. RR provided the conceptual input, wrote, edited, and provided funding for the manuscript. All authors contributed to the article and approved the submitted version.

## Conflict of Interest

The authors declare that the research was conducted in the absence of any commercial or financial relationships that could be construed as a potential conflict of interest.
